# Heart Beats

**Published:** 1885-03

**Authors:** 


### Heart Beats.

Dr. N. B. Richardson, of London, says he was recently able to convey a considerable amount of conviction to an intelligent scholar by a simple experiment. The scholar was singing the praises of the "ruddy bumper," and saying he could not get through the day without it, when Dr. Richardson said to him: "Will you be good enough to feel my pulse as I stand here!" He did so. I said: "Count it carefully, what does it say?" "Your pulse says 74." I then sat down on a chair and asked him to count it again. He did so, and said, "your pulse has gone down to 70." I then laid down on the lounge and said: Will you take it again?" He replied: "Why, it is only 64, what an extraordinary thing!" I then said: "When you lie down at night, that is the way nature gives your heart rest. You know nothing about it, but that beating organ is resting to that extent, and if you reckon it up it is a great deal of rest, because in lying down the heart is doing ten strokes less a minute. Multiply that by sixty and it is six hundred; multiply it by eight hours, and within a fraction it is five thousand strokes different, and as the heart is throwing six ounces of blood at every stroke, it makes a difference of thirty thousand ounces of lifting during the night. When I lie down to rest without any alcohol, that is the rest my heart gets. But when you take your wine or grog you do not allow that rest, for the influence of alcohol is to increase the number of strokes, and instead of getting this rest you put on a difference of something like fifteen thousand extra strokes, and the result is you rise up very seedy and unfit for the next day's work until you have taken a little more of the "ruddy bumper" which you say is the soul of man below.—*Gaillard's Journal.*

